# Improving Mechanical and Thermal Properties of Cellulose Foam with Alumina Nanofibers

**DOI:** 10.3390/polym17081043

**Published:** 2025-04-11

**Authors:** Sirje Liukko, Katarina Dimic-Misic, Aleksandar Janackovic, Michael Gasik

**Affiliations:** 1 Department of Chemical and Metallurgical Engineering, School of Chemical Engineering, Aalto University, 02150 Espoo, Finland; sirje.liukko@aalto.fi (S.L.); michael.gasik@aalto.fi (M.G.); 2 Institute of General and Physical Chemistry, 11000 Belgrade, Serbia; ajanackovic@iofh.bg.ac.rs

**Keywords:** cellulose foam, alumina nanofibers, cellulose composite, foam rheology

## Abstract

Foam-formed cellulose biocomposites provide a promising, innovative approach to creating lightweight and eco-friendly materials for utilization in packaging and insulation. This study investigates the production and characterization of temperature-resistant, mechanically stable cellulose fiber (CF) composite foams reinforced with alumina nanofibers (ANFs). To evaluate the impact of ANFs on rheology and drainage, CF suspensions were prepared at a concentration of 20 g/kg, with ANFs added at 2 wt% and 5 wt%. All foams exhibited shear-thinning behavior, with variations in flow characteristics influenced by ANF consistency and particle–bubble interactions. ANFs were integrated into the dry CF foam structure using two methods: (i) immersion in an ANF water suspension, and (ii) direct injection of the suspension into the foam matrix. Mechanical and thermal analyses of the dried CF foams with 2% ANFs demonstrated significant improvements in strength and thermal stability. Incorporating ANFs into CF-based foams enhances their rheological properties, improves mechanical and thermal performance, and reduces combustion rates. These results highlight the potential of ANF-reinforced CF foams for use in industries requiring biodegradable insulation and packaging materials.

## 1. Introduction

Stricter regulations and growing concerns over the environmental impact of fossil-based materials have sped up the advancement of sustainable packaging.

As a manufacturing process, the foam-forming process enables the formation of complex shapes and tailored structures of material matrix, which can affect a material’s properties and enhance its performance. In composite materials, foam forming allows control of the porosity of materials in a composite and their cellular structure. Foaming techniques are then applied to create the desired cellular structure within the biocomposite material, which can be achieved through physical, chemical, or biological methods. Physical foaming involves using compressed gases or volatile liquids, which expand upon pressure release or temperature increase, creating voids within the matrix. Chemical foaming, on the other hand, relies on chemical reactions that generate gases, such as carbon dioxide or nitrogen, in situ, leading to the formation of cellular structures. Mycelium composites represent a particularly interesting class of biocomposites, utilizing the filamentous hyphae of fungi to convert low-cost organic waste into materials with potential applications in acoustic damping, super absorbents, and even structural components.

A common packaging and isolation material is expanded polystyrene (EPS), whose recycling is limited and complex, requiring several steps for segregation from other materials. As a result, developing eco-friendly alternatives to plastic foams is a shared goal within the scientific community [1]. The utilization of lignocellulosic or natural fibers such as jute, hemp, bamboo, sisal, and wood fibers as reinforcement agents in liquid foams and biocomposites has gained prominence due to their ability to produce highly bio-compostable material.

Cellulose, the most abundant biopolymer on Earth, is primarily sourced from woody plants, making it a significant and renewable resource. Being a biopolymer, cellulose is biodegradable and possesses exceptional mechanical properties. Thanks to advancements in chemistry, it can be processed into various forms, such as foams, films, and aerogels, at the same time preserving its strength and biodegradability [2].

Cellulose fiber-reinforced composites, produced via foam-forming techniques, represent a fast-expanding area of materials science. They have gained significant attention due to their potential for creating lightweight, sustainable, and high-performance materials. These composites combine the advantages of cellulose fibers, such as their high specific strength and stiffness, biodegradability, and abundance, with the unique characteristics of foam structures, including a low density, high surface area, and excellent thermal and acoustic insulation properties. In this context, current research with cellulose and its derivatives is related to the production of packaging materials and the development of new composite materials that can take the place of fossil-based polymers, and mitigating environmental pollution caused by plastic packaging. In response to this need, cellulose has been described as a flexible and abundant raw material that is suitable for the foaming process, in which the mixing of fibers, surfactants, and water achieve the required air content [3,4,5] to overcome problems related to the incomplete shock absorbance of existing molded pulp cushions; but the developed novel biopolymer foams have poor recyclability.

Recently developed biopolymers based on fibrous foams can provide materials with improved mechanical properties and solutions to cushion structures utilizing raw materials from the paper industry. Furthermore, these foam products are fully biodegradable, with over 90% degradation within a period of three months [6]. Combining cellulose fibers and foam-forming techniques utilizing aqueous foams allows for the creation of materials with a wide range of properties, tailored to specific applications.

Liquid foams are colloidal systems where a discontinuous gas phase is dispersed throughout a continuous liquid phase. This dispersion results in numerous gas bubbles that can either pack closely together, forming the distinctive cellular structure of the foam, or remain suspended freely in the liquid, producing confined foam [7]. Confined foam is restricted by its boundaries, such as a porous medium, while bulk foam exists freely without such constraints [8]. The distinct cellular configuration determines a foam’s formation and flow, with properties that differ from those existing in its constituent gas and liquid phases, stabilized by surfactants [9]. There are two types of foam: (i) wet foam, containing a higher liquid content, making it more fluid and unstable, containing thicker liquid films between gas bubbles; and (ii) dry foam, containing liquid in its matrix, resulting in thinner films and a more stable, solid-like structure [10,11]. Wet foam is often seen in freshly poured beverages while dry foam is more common in shaving creams or foamed plastics; during drainage of the foam, a change occurs from wet to dry foam, producing changes in the foam’s stability and texture [12].

In the 1970s, the paper industry witnessed a significant breakthrough in introducing foam-forming technology [13,14]. This innovative approach offered cost-efficient and sustainable alternatives to traditional paper production methods, allowing for the incorporation of various raw materials [15]. Dewatering, a crucial step in the paper-making process, was a particular area of focus for researchers exploring foam forming. Studies revealed that while foams have higher viscosity than water, their dewatering is much higher [16,17].

In the foam-forming process, aqueous foam is used as a vehicle for the transportation of cellulose fibers and their deposition onto desired points of the material [18,19]. Foam-forming methods were developed for the needs of the non-woven-textile industry for the transportation of technical fibers [20,21]. After a quiet period, foam-utilization processes, such as using foams for coating and the layering of fibers and particles, are now attracting increasing interest in various industries [22,23]. Over the last ten years, there has been increased interest, resulting in a large number of publications related to foam products made from raw materials such as cellulose fibers, nanocelluloses, and cellulose derivatives [24,25]. Products produced from cellulose foam exhibit various favorable properties such as antimicrobial properties, mechanical anisotropy [5], thermal stability [26], and hydrophobicity [27]. One of the important aspects of foam forming is drainage, including the effect of the gravity-driven downward flow of liquid, the coarsening effect (where small bubbles combine with bigger bubbles caused by pressure-driven gas diffusion), and the timeframe for bubble coalescence [28]. Foam drainage is critical for the preservation of the stability of foam, as during drainage the liquid moves through a bubble matrix in both wet and dry foams with increased freedom of movement of bubbles, with the process easily viewed as bubbles’ vertical movement towards the surface of foam, driven by hydrostatic pressure [29,30]. In the case of dry foam, the liquid layers between the bubbles grow thinner and can rupture, resulting in the collapse of the foam structure [31,32].

Possible mechanisms affecting the mechanical properties of cellulose foam are rheological behavior and failure of foam materials which are non-uniform joints due to fiber drainage and fiber bending, buckling, or inter-fiber bond opening [33,34,35]. Inter-fiber bond failures may also happen once material is compressed, and they have been reduced by increasing the elasticity of bonds with the addition of polymers [36,37]. Therefore, the addition of fillers, fibers, and polymers has been utilized to improve the rheological and mechanical properties of cellulose foams [38,39]. Due to problems related to the aging of liquid foam and wall slip during motion past solid boundaries, their rheological evaluation is complicated and requires special geometries that reduce viscosity inhomogeneities across the flow [40].

In the context of improvement of the properties of cellulose-based biocomposites, various nanofibers may be promising alternatives due to their high surface/volume ratio, which brings a high anisotropy index [41]. In the flow of fibers with a simple structure aligned in one dimension, upon dewatering, the formed structure has improved mechanical properties and the possibility for application as filters or reinforcing phases. Among the variety of ceramic nanofibers, those made from the oxidation of aluminum, alumina nanofibers, have ultra-high temperature resistivity and chemical stability [42,43,44,45]. Alumina (Al_2_O_3_) particles are widely utilized across various fields, including as adsorbents, composite materials, and in membrane preparation, due to their high surface area, large pore volume, and significant porosity. Alumina nanofibers (ANFs) may offer a promising inorganic component as they can add to the formation of composite structures, which mimics the hierarchical structures found in nature. Such aligned oxide nanofibers, with their unique slit-shaped pore structures formed by parallel nanofiber gaps, have shown potential in applications such as membranes and thin-layer chromatographic media [42,43,44,45]. Aluminum oxide (Al_2_O_3_) exists in several polymorphs, such as metastable α, β, and η phases as well as thermodynamically stable γ-Al_2_O_3_. The metastable (also known as transition) phases of alumina are intrinsically nanocrystalline in nature. They can be synthesized by various methods including hydrothermal, sol–gel processing, chemical vapor–liquid–solid deposition, and electrospinning routines. Transition aluminas, especially γ-Al_2_O_3_, are widely produced as catalyst carriers in the automotive and petroleum industries, structural composites for spacecraft, abrasive and thermal wear coatings, and filter applications; gamma-alumina (γ-Al_2_O_3_) is reported to occur over a wide interval at temperatures between 350 and 1000 °C and is typically obtained from an amorphous or boehmite precursor. The properties of the final alumina products are highly dependent on the crystalline structure, morphology, and microstructure of the polymorph; therefore, many research efforts have focused on the study and characterization of transition aluminas with respect to their transformation mechanisms, changes in porosity, specific surface area, surface structure and chemical reactivity, and the defect crystal structure. Reports on the thermal treatment demonstrate that the thermal behavior depends essentially on the phase composition and morphological parameters of the precursor material (usually boehmite AlOOH), such as degree of crystallinity, size of crystals, porosity, etc. The morphology and size of the final Al_2_O_3_ strongly depend on the precursor used during the conversion process, synthesis method, and defect content of the crystal. The recently developed controlled liquid-phase oxidation of liquid aluminum enables the production of alumina nanofibers (ANFs) with extremely high aspect ratios, exceeding 10^7^, and diameters ranging from 5 to 50 nm. Crystal growth from a metal melt is an effective method for producing nanomaterials due to its low cost, high yield, and ability to achieve high-purity oxide structures. In this study, γ-Al_2_O_3_ nanofibers with extreme aspect ratios obtained from oxidation of liquid aluminum are used [42,46]. Formation of these vertical nanorods occurs at the transition from the gamma to the alpha phase at exceptionally high temperatures of 1200–1250 °C [42,46].

Furthermore, their application improves the mechanical properties of the composite and increases the melting point. These nanofibers are chemically hydrated (2–6 wt%) gamma-alumina (γ-Al_2_O_3_)-phase fibers with a high aspect ratio (length-to-diameter ratio) of around 10^7^ [46].

We postulate that incorporating ANFs into the cellulose foam (Table 1 and Table 3) can enhance its sustainability and improve the processing flow behavior while also contributing to the thermomechanical stability of the composite material in its dry state. Moreover, we hypothesize that ANFs will further improve the rheology of the liquid cellulose–alumina foam and enhance the mechanical performance of the final composite product. Therefore, the objectives of this study are threefold: (i) to report innovative composite foams based on cellulose and ANFs; (ii) to present the complexity of the foam manufacturing process, including methods such as mixing and injecting or immersing ANFs into the foam; and (iii) to evaluate the rheological, mechanical, and thermal characteristics of the resulting composite foams.

## 2. Materials and Methods

### Materials

Fibers (cellulose and alumina): For the preparation of cellulose foam, cellulose fibers (CFs) were obtained from pulp produced from never-dried bleached birch from a Finnish pulp mill (Stora Enso Oyj, Helsinki, Finland), with an average fiber length of 1.23 mm; they were obtained with a FibreLab analyzer (Metso Automation GmbH, Düsseldorf, Germany). Nanostructured alumina ceramics are gaining attention for their enhanced strength, with one promising production method involving the oxidation of molten aluminum in an oxygen-rich environment. This process forms alumina nanofibers upon the reaction of aluminum with oxygen, and the properties and thickness of the fibers are influenced by factors like metal purity, oxygen flow, and reaction conditions [41]. Commercially available ANFs [41] of ~40 nm diameter and length 5–6 cm with a specific surface area of 155 m^2^/g, determined from an optical camera image and SEM image (Figure 5c,d), were partially cut to be mixed as nanofiber bundles with the cellulose as described below.

Foam: Liquid foams were produced by mixing tap water with the anionic surfactant, sodium dodecyl sulphate (SDS, C_12_H_25_SO_4_Na), 8.5 mM, which is above the critical micelle concentration of the volumetric liquid fraction of 0.2%. Foam that had cellulose fibers was produced by pouring cellulose pulp into the SDS solution. The molar concentrations of the SDS solution and CFs used in this study are presented in Table 1. The half-life of the foam was measured offline using samples from the foam mixing vessel, and it varied between 4 and 5.5 min.

**Figure 3:**
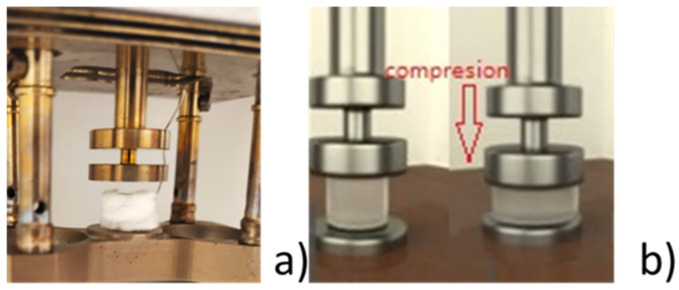
Mechanical testing: DMA set up with foam sample being placed on the fixed bottom plate and the upper plate making contact with the upper surface of the sample. (**a**) Foam sample placed on instrument; (**b**) schematic image of a creep compression test.

CF-laden foams were prepared by adding hardwood fibers at 20 g/kg fiber consistency to the 2 wt% SDS solution, calculated from the amount of dry cellulose. These CFs had long, slender structures, with a characteristic length around 20 times the mean bubble radius and average length of ~17 µm (30–40% of the mean bubble radius), and coarseness 0.1 mg/m. The protocol to fabricate low-density foam consists of several distinct steps, presented in Figure 3, using the already described method [46]. Initially, CFs were mixed into a pulp suspension and poured into a suspension of surfactant and water and mixed with vigorous mixing (Netzsch Drill Pulp Agitator, Hedensted, Denmark). The aqueous CF foam was ejected with a pressure pipe into a rectangular metal wire, porous at the bottom side. Water was drained from the foam matrix for 60 min, and after 3 h the formed foam sheet was, together with the wire, placed into an oven at 70 °C overnight. Afterward, the dried foam sheet was sprayed with water to 50% dry solids content and pressed to obtain a final material density of 40 kg/m^3^.

Sample preparation: For rheological evaluation, samples were prepared as liquid aqueous foams with hardwood fibers, as reference foam, and foams that, apart from cellulose fibers, had two different concentrations of ANFs, with the constituent ratios presented in Table 2. Table 3 presents the experimental methods used for the evaluation of the liquid and dry foams, with the formulations presented in Table 2.

The main methods for the input of traditional fire-retardant solutions were simulated through co-foaming, injection, or coating. Hence, we used three different approaches to bring the cellulose fibers into contact with the ANFs. The concentration of SDS was 2 wt% while the amount of ANF was 5 wt% of the dry cellulose fiber weight, as presented in Table 2. The experimental analysis was performed according to the type of sample from Table 1 and Table 2, as presented in Table 3.

**Table 3:** Schematic presentation of measurements performed on liquid and dry foams and volume/dimensions of samples.

Liquid Foam Sample	Sample Amount/mL	Number of Samples	Dry Foam Sample	Sample Dimensions/mm	Number of Samples
Drainage	1500	3	DMA dry	10 × 10 × 10	3
Rheology	15	5	DMA wet	10 × 10 × 10	3
Optical imaging	2	3	STA dry	3 × 3 × 4	3
Camera imaging	1	2	SEM	1 × 1 ×1	3

The experimental setup of this research follows two distinct protocols based on the presence of liquid in the samples. Initially, foam was analyzed for its drainage and rheological properties. In the second stage, the dried foam was impregnated with ANF and evaluated for its morphology, mechanical properties, and thermal stability.

**Figure 1:**
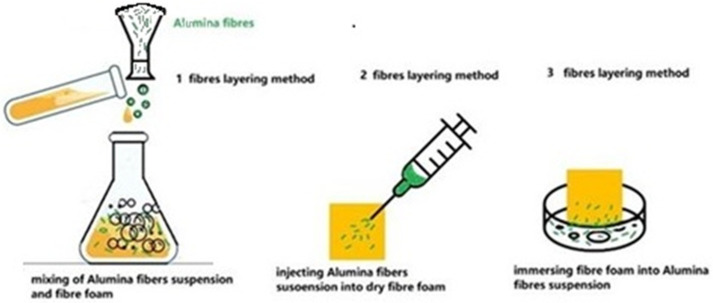

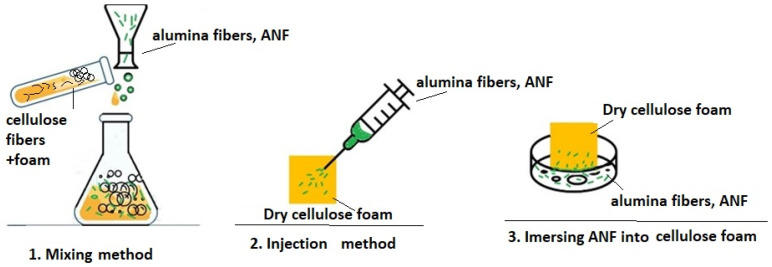
Schematic presentation of the method used for the preparation of the dry composite foam with ANF fibers: mixing of cellulose suspension with ANF fibers, injecting ANF fiber suspension into dry foam samples, and immersing dry cellulose foam samples in a flask with ANF suspension.

The first method of preparation of a cellulose–ANF dry foam composite consisted of an ANF suspension being poured simultaneously with a cellulose foam suspension into the flask, and the mixture was drained for 2 h and dried in the oven. In the second method, the ANF suspension was injected with the syringe of an 0.8 mm radius needle into dried fiber foam. Injection of the ANF suspension occurred at several points from all sides of the foam. In the third method, dried fiber foam was placed in a glass vessel containing the ANF suspension, and foam emerged from each side for 30 min, enabling capillary penetration of the ANF suspension to suck the liquid and possibly some nanofibers into the foam pores or leave them on the surface of the foam, as schematically presented in Figure 1.

## 3. Characterization of the Samples

Rheometry: Rheological experiments were made with an MCR 302 rheometer (Anton Paar, Graz, Austria) with the use of the Couette geometry in order to minimize the presence of wall slip of the foaming suspension and reduce mistakes obtained in the measurements. Steady-state measurements were performed to determine the variation in the dynamic viscosity (*η*). The geometry used was the “bob in cup” geometry, where the inside of the cylinder, which was a metal cylinder with a diameter of 17 mm, was placed in a serrated, four-bladed vane spindle with a diameter of 10 mm and a length of 8.8 mm (Anton Paar, Ostfildern, Germany). The dynamic flow curves of the gel-like foam suspension were evaluated when increasing the shear rate range ($\dot{\gamma }$ = 1000–0.01 s^−1^). The data duration had a logarithmic spread in the interval from 1 to 100 s. The Ostwald–de Waele expression for purely viscous shear thinning is given by

(1) $$\eta =k{\dot{\gamma }}^{n-1}$$

where *k* and *n* in Equation (1) are the consistency and power law index, respectively, and $\dot{\gamma }$ is the shear rate.

To ensure the same measuring protocol for each sample, before each measurement the samples were pre-sheared at a constant shear rate of $\dot{\gamma }$ = 100 s^−1^ for 60 s and left to rest for another 60 s.

In order to reach the viscoelastic structure of the foams, viscoelastic measurements were conducted within the linear viscoelastic region (LVE) and evaluated with an amplitude sweep SAOS test, where strain (*γ*) was in the range between 0.1 and 500%, at a constant angular frequency (*ω*) of 10 rad·s^−1^, to determine the effective linear viscoelastic region (LVE). After the SAOS tests were conducted, frequency sweep measurements were performed at strain within the LVE of *γ* = 0.1% angular frequency (*ω*) in the range of 0.1–100 rad·s^−1^. The dynamic viscoelastic moduli were observed, and the changes in the storage modulus (G′) and loss modulus (G″) were followed as a function of strain amplitude (γ) and angular frequency (*ω*). The ratio of the loss modulus (G″) to the elastic modulus (G′) is the damping factor (tan(δ) = G″/G′), and the change from a liquid towards a viscoelastic solid was observed in temperature ramps from 10 to 70 °C. The Herschel–Bulkley equation describes the presence of a dynamic yield stress (*τ*_d_^0^) from the plot of the flow curves as [38,39,40]

(2) $$\tau_{d}=\tau_{d}^{0}+k{\dot{\gamma }}^{n}$$

As strain (γ) is constantly increased at a constant frequency (ω) from oscillatory amplitude sweep measurements, the maximum in the elastic stress (τs) that corresponds to the static elastic yield stress (τs0) is determined as the first point of deviation from linear elastic deformation, occurring at a corresponding critical strain value γc. The elastic stress component in the oscillatory mode is given by

(3) $$\tau_{s}^{0}=G^{\prime }\gamma_{c}$$

Data variation in rheological measurements of cellulose suspensions, hydrogels, and foams is within 10% due to the thixotropic nature of such hydrogels accompanied with shear banding phenomena [38,39].

**Figure 2:**
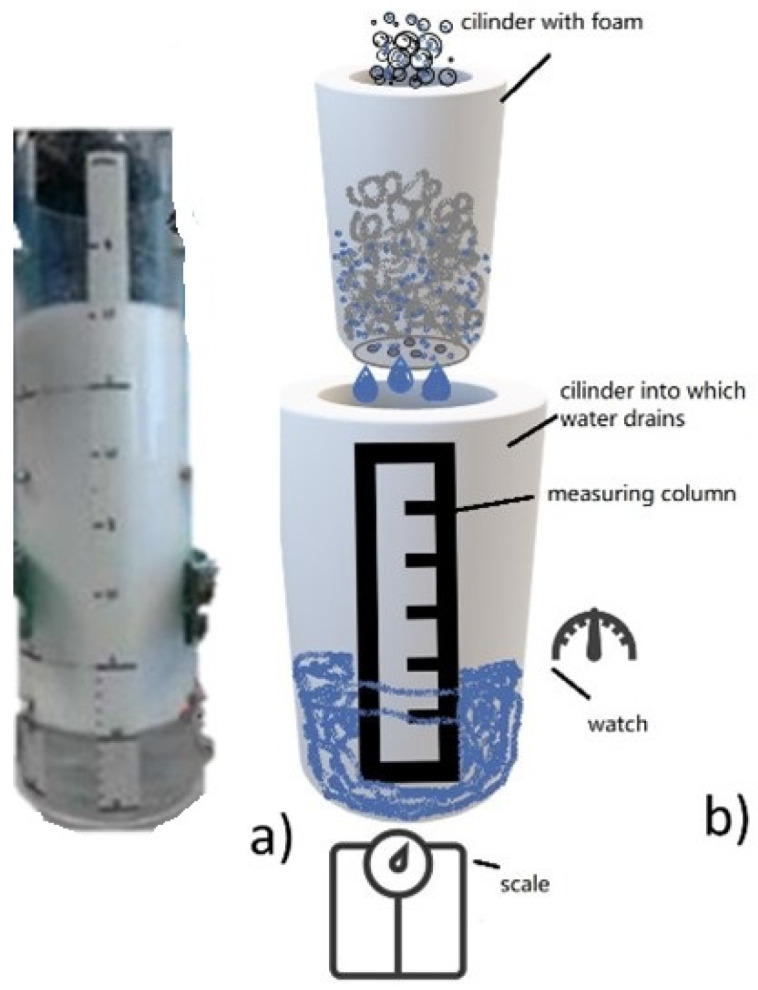
Experimental setup of drainage of a cylinder with foam suspension being poured into a cylinder with a porous bottom and a second cylinder into which liquid drains; (**a**) cylinders for foam and collection of liquid; (**b**) schematic drawing of the experimental setup.

Drainage: Drainage of foam suspensions is extremely important when fiber foams are used as it affects the stability of the liquid foam. Improving the stability of wet foams requires knowledge of the mechanisms and factors that affect drainage. To measure the drainage of the cellulose foam, the foam was poured into a cylinder with a porous bottom and diameter of 165 mm, which was placed on a stand above another graduated cylinder, as presented in Figure 2. The water runoff was collected in the outer cylinder and its mass difference, as the liquid from the inner cylinder liquid was drained, was recorded. The timer was initiated and regularly recorded the amount of liquid collected inside the cylinder, and presented the drained liquid, until the drainage rate stabilized, measuring the volume of the liquid at consistent intervals. The measurements were made at a given volume of drained water, providing data on how quickly the liquid drained from the foam over time.

Mechanical analysis: Mechanical testing was performed using a dynamic mechanical analysis (DMA) with the “Artemis 242E” equipment (Netzsch Gerätebau GmbH, Selb, Germany) with creep or pseudo-static settings. Compression mode was used for testing, with samples being in the dry and wet states. All samples had an approximate size of 10 mm × 10 mm × 10 mm, where wet samples were made by placing a 1 mL drop of water on the foam sample to ensure complete wetting. The sample holder was calibrated on an empty system stiffness with static, dynamic, and rotation setups, and the results were subtracted with a resolution of ±0.5 nm (Figure 3a). The creep test exposes the sample to a static load in a continuous manner over a specific period, and the obtained deformation is recorded, as presented in Figure 3b. All samples were loaded twice in these tests: first with 0.1 N and second with 0.5 N loads, with recovery phases in between. These phases are called loading 1/unloading 1 and loading 2/unloading 2, respectively. For all tests, variations in length, stiffness, static stress/strain ratios, creep compliance, modulus, and other properties were evaluated.

**Figure 4:**
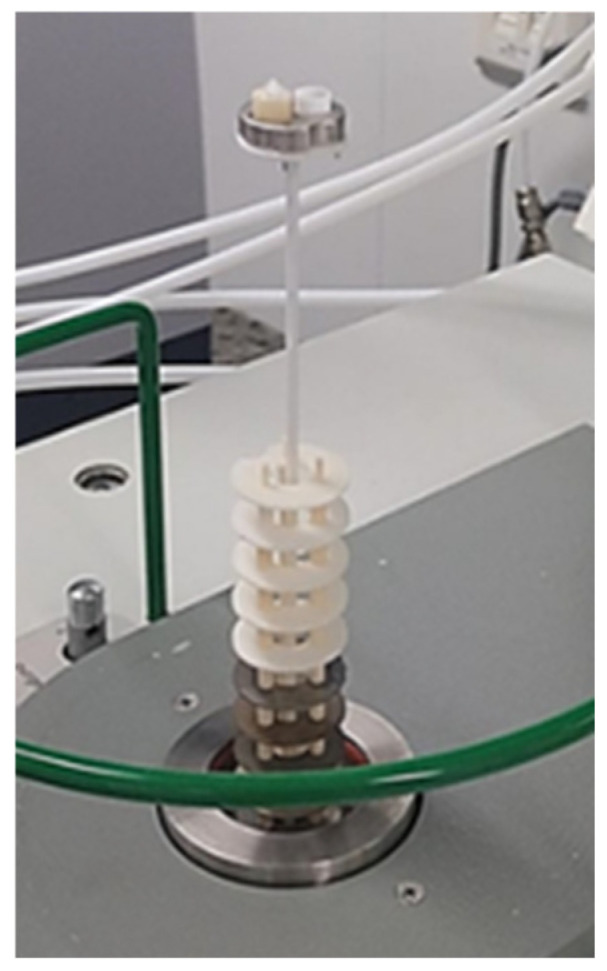
STA experimental setup with two crucibles; one was empty and the other contained foam samples.

The thermal behavior of the samples was analyzed using a simultaneous thermal analysis (STA) apparatus STA449C “Jupiter^®^” (Netzsch Gerätebau, Germany). The foam samples were heated in alumina crucibles without lids, with an airflow of 10 mL/min and a heating rate of 10 °C/min, to 700 °C. As a DSC reference, an identical empty alumina crucible was used. A baseline measurement with empty reference and sample crucibles was run as well to subtract the influence of the empty crucibles and the sample holder, as depicted in Figure 4. From the DSC runs, the onset, peak and end temperatures, and enthalpies were determined.

Imaging: The surfaces of the foams were inspected with an optical microscope BX53M with camera DP74 (Olympus, Tokyo, Japan). Optical microscopy depicted the formation of air bubbles formed in the foams’ surface layers. Additionally, scanning electron microscopy (SEM) was used to examine cut segments of different foam blocks to observe the cross-section structure of the bubbles. Electrical conductivity was improved by coating samples with a thin layer of Au/Pd 80, and afterwards samples were mounted on carbon tape.

## 4. Results and Discussion

**Figure 5:**
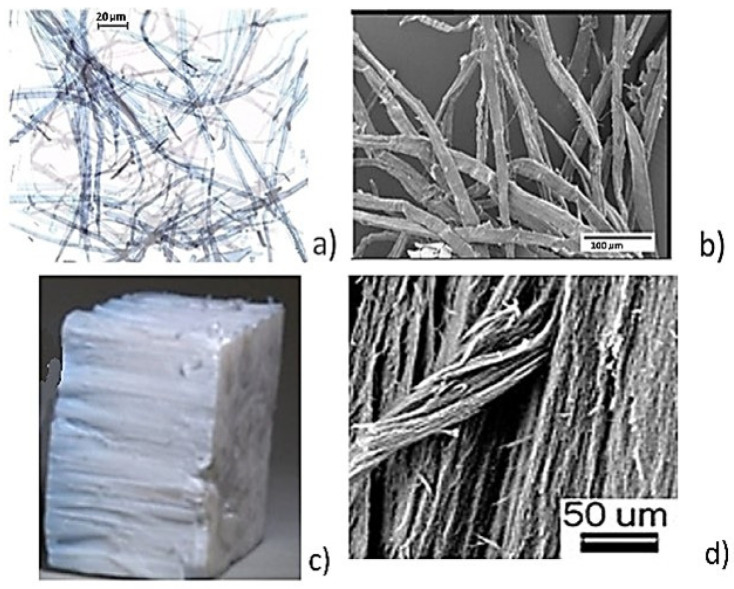
Birch hardwood bleached kraft pulp images: (**a**) optical image and (**b**) SEM image. (**c**) Example of stack of alumina nanofibers (ANFs) and (**d**) SEM image of ANFs.

In Figure 5, an optical microscope micrograph and an SEM micrograph are used to present the morphology of the fibrous materials used in this study. It is seen that the cellulose fibers are branched and flocculated with an uneven surface. In their contacts, the alumina nanofibers are organized in a brick-type white structure in which they are firmly bound to each other, and oriented upwards with a smooth surface (Figure 5c,d).

**Figure 6:**
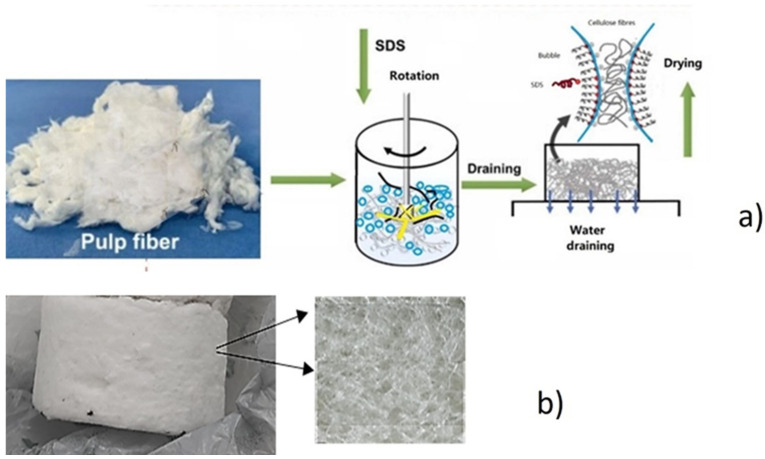
Schematic presentation of the (**a**) foam production process from cellulose pulp towards mixing CF with SDS and water and finally drainage on a porous wire mesh, and (**b**) dry foam with high porosity.

**Figure 7:**
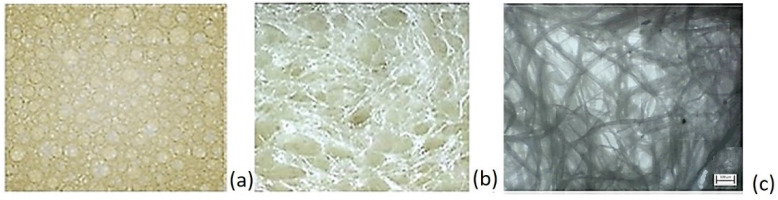
Images of cellulose fiber (CF) foam: (**a**) liquid foam, (**b**) macroscopic image of dry foam with fibers, and (**c**) SEM images of dry foam. A porous structure with small and large pores is characteristic of cellulose foam morphology.

The method for the preparation of the foam samples consisted of three major stages: the first was the preparation of cellulose suspensions from dry cellulose; the second was pouring the cellulose suspension and surfactant SDS into the foaming device with vigorous mixing; and the third was drainage of water and drying of the foam, as presented in Figure 6a. The final product of this foam production process was a white porous light foam, which has a porous structure (Figure 6b and Figure 7b).

Optical images of the liquid foam, presented in Figure 7, and dried samples of the cellulose fiber foam, presented in Figure 7b, show the bubbles surrounded with cellulose fibers and the extent to which the structure has been modified by the foam bubbles and the formation caused by the bubble meniscuses where fibers were around the bubble surface, causing internal stress during mixing. In the images in Figure 7b,c regions where the CF matrix structure was replaced by large voids, which were air bubbles, can be observed. There are differences in the sizes of openings within the CF structure due to differences in bubble size. When the suspensions are in the liquid form, large voids correspond to formation by bubbles, while smaller ones correspond to water pockets formed between fibers. Such structural arrangements regarding the bubble size are crucial in facilitating foam drainage—a phenomenon examined in detail later.

**Figure 8:**
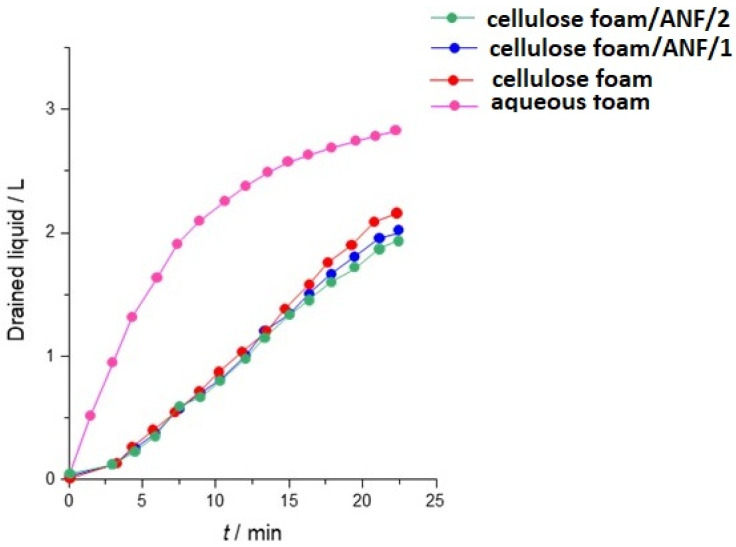
Drainage rate of water from liquid foam suspensions measured with cylinder method. The presence of cellulose fibers improves drainage, slowing down dewatering, and furthermore, the presence of ANFs adds to better dewatering.

As mentioned earlier, foam formation is a complex hydrodynamic process that involves an intricate balance between various physical and chemical factors. A critical aspect of this process is the efficient drainage of water from the foam structure, which is essential for the formation of dry and stable foam. Drainage is important to be slower for the uniform movement of meniscus downwards, and Figure 8 presents the difference in drainage rates between aqueous foam and aqueous CF suspensions, with and without ANFs. A decrease in the CF drainage rate is favorable, as the transition from wet to dry foam is more uniform and therefore favorable for a more uniform dry foam structure and porosity, affecting the formation of the foam. The addition of CFs to the foam matrix decreases the drainage rate, and the addition of ANFs does not change the drainage rate further. A slight decrease in drainage rate was observed with the addition of ANFs, and an increase in their consistency further decreased the drainage rate.

**Figure 9:**
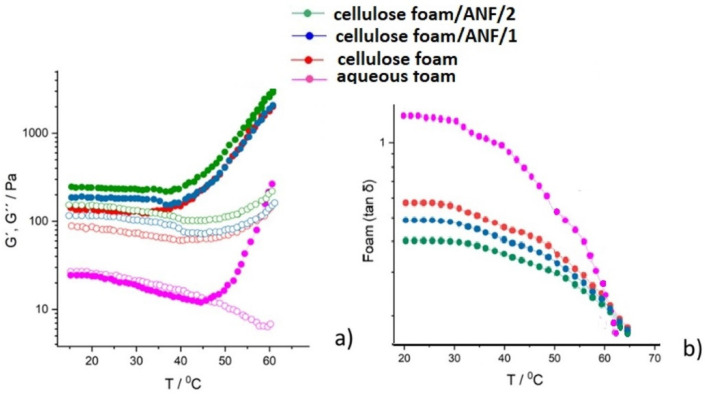
Temperature ramps during amplitude sweep tests obtained for the aqueous foam, CF foam, and CF alumina foam suspensions: (**a**) storage and loss modulus as a function of temperature and (**b**) the loss factor tan(δ) as a function of temperature. Closed symbols Storage Modulus (G′) open symbols Loss Modulus (G″).

With increasing temperature, liquid evaporates from the foam and bubbles collapse, making the transition to dry foam, which becomes a suspension of higher solids content and dry foam. This can be seen in Figure 9a through the change in rheology as there is a transformation from a more viscous toward a more viscoelastic structure, an increase in G′, and a decrease in G″. Correspondingly, the decrease in G″ values is more drastic for the mixture of the foam suspension with ANF in the reference cellulose-only suspension. Similarly, all ANF-containing suspensions have a more uniform transition from the liquid to the solid phase, without a distinct edge, showing a sharp transformation from the liquid towards the dry foam phase with increased temperature. Similarly, Figure 9b presents the change in tan(δ), with the highest values at low temperatures, when the liquid nature of the foam dominates, with a different rate of decrease as the temperature increases and the liquid phase evaporates. Only aqueous foam has a more drastic decrease in tan(δ) than foams that contain CFs and ANFs. The addition of ANFs smoothens the transition between the liquid and solid state due to drying, which is favorable for foam formation and uniformity of the foam matrix.

**Figure 10:**
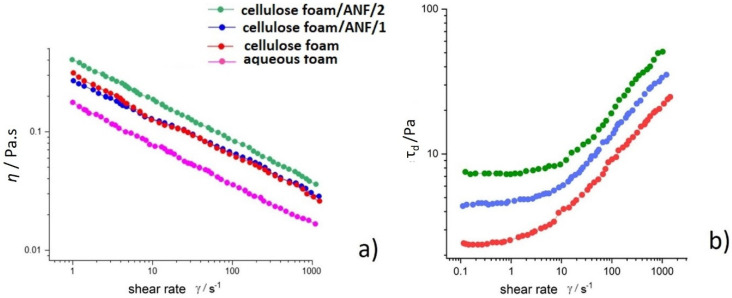
Dynamic steady-state curves of foam suspensions: (**a**) dynamic viscosity (η) as a function of shear rate $\dot{\gamma }$, and (**b**) corresponding dynamic stress (τ_d_) dependence on shear rate.

From the steady-state flow curves, it is evident that ANF fibers do not interact between themselves and do not flocculate, which is in contrast with long and branched CFs. The bubble curtain surrounding the fibers prevents direct collisions and flocculation during the suspension flow. However, all four foam suspensions, (i) surfactant–water foam, (ii) cellulose fiber foam, and (iii) two cellulose fiber with ANF foams with different concentrations of alumina, show shear thinning behavior, presented in Figure 10a; and the dynamic stress (*τ*_d_) increases with the addition of ANF fibers to the CF suspension (Figure 10b). Fits of the flow curves using Equation (1) for the same shear thinning index *n* = 0.6, with a limit of error in the given ranges, gave a value of *n* = 0.61 ± 0.03 for the five measurements. The fitted values of the consistency index *k*_0_ when *n* = 0.6 are presented in Table 4. The dynamic stress (*τ*_d_) of the flow curves is shown in Figure 10b.

The viscous performance of foams is generally described with a power law, and inclusion of AFN particles did not change this general behavior. Moreover, in all the cases, the flow behavior index was the same, namely, *n* = 0.6. The presence of cellulose fibers increased the viscosity considerably compared to that of pure foam, whereas the addition of ANFs had a minimal effect on the foam viscosity. The difference in the strength of interaction between the fibers and bubbles could explain this difference. Cellulose microfibers also increased the viscosity, but their effect was smaller than natural fibers. While also interacting strongly with bubbles, particles are much smaller, and the momentum transport is not as efficient as with cellulose fibers. The dynamic viscosity (*η*) obtained from Equation (1) and the yield stress values τ_d_^0^ obtained from Equation (2) are presented in Table 4. As expected, the *τ*_d_^0^ increases with the addition of ANF fibers; this is obtained when flow curves are fitted to a Herschel–Bulkley model (Equation (2)). Similarly, fitting steady-state data to Equation (1) with *n* =0.6 provides results for the parameter k. The static yield stress, obtained from Equation (3), for strain sweep measurements obtained with constant temperature, for *T* = const in the range 10–70 °C, is calculated as tan*δ*_min_, where G′ >> G″, and where the foam reaches the transition from a liquid towards a viscoelastic solid (Table 4).

**Figure 11:**
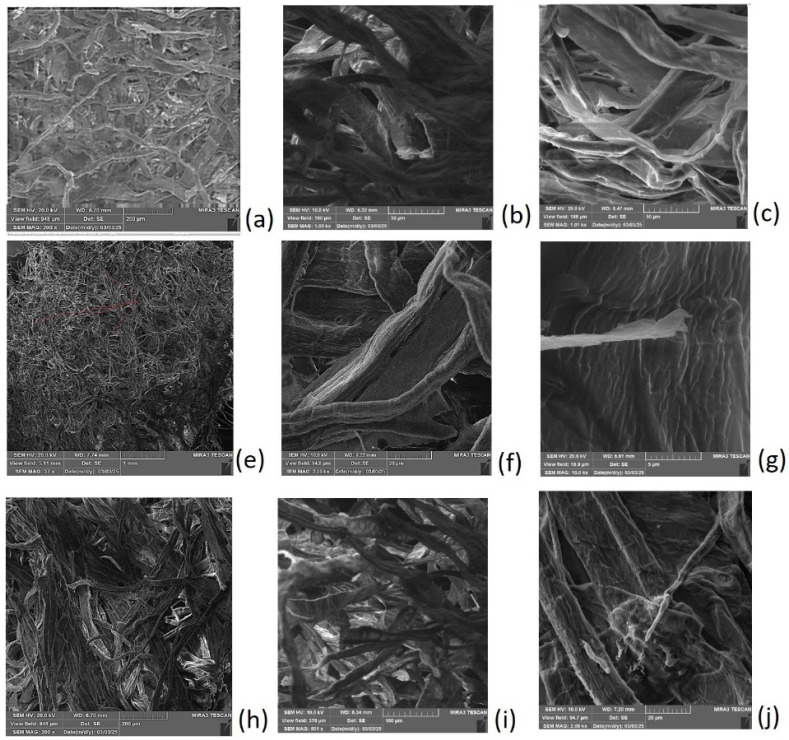
Empty foam without alumina fibers (**a**–**c**); and images where ANF fibers are visible as straight, white lines formed by injection with an ANF suspension, (**e**–**g**) and foam immersed into an ANF suspension (**h**–**j**).

The flow properties of aqueous foam suspensions have also been studied previously, and in this study, we observed that for n = 0.6 the factor of two is the value at which the addition of CFs increases the *η* of the foam. In the SEM micrographs (Figure 11), there are dry cellulose foams, empty foams that are used as a reference sample (Figure 11a–c), an injected foam (Figure 11e,f), and immersed foams (Figure 11h–j). It is clear that all the foams are made of fibers organized into a distinct open porous structure. Voids between the fibers are formed after the drainage and evaporation of water from bubbles. The dried foam samples reveal the extent to which bubble formation and meniscus effects influence the structure, as the fibers make contact through aligning along bubble surfaces, forming hydrogen bonding during drying. There are larger and smaller voids corresponding to a bigger open structure that corresponds to the locations of former bubbles, while smaller voids correspond to regions where smaller bubbles or water pockets formed between cellulose aggregates.

At the highest magnification in Figure 11a, both large and small voids within the cellulose fibers are visible, contributing to a uniform porosity distribution throughout the foam sample. The imaging effectively distinguishes the void structures, revealing how air bubbles mix within the foam suspension, causing fibers to align along meniscuses and aggregate into a fibrillar network. When cut, connected ANFs come into contact with the cellulose foam, either attaching to fibers or resting against two neighboring fibers.

The surface area of the ANFs is significantly smaller than that of the fibers, appearing as thin white rods (Figure 11e–h), or as short white sticks when bundled (Figure 11f,g). Since the ANFs used in this study are derived from larger, oriented structures, they primarily consist of multiple fibers firmly bound together. In SEM micrographs, they appear as sharp, straight, and bright white objects. The difference between the empty foam and the foams with immersed and injected alumina fibers is evident, as very small white alumina rods are attached to the cellulose fibers, distinguishing the modified foam structure.

**Figure 12:**
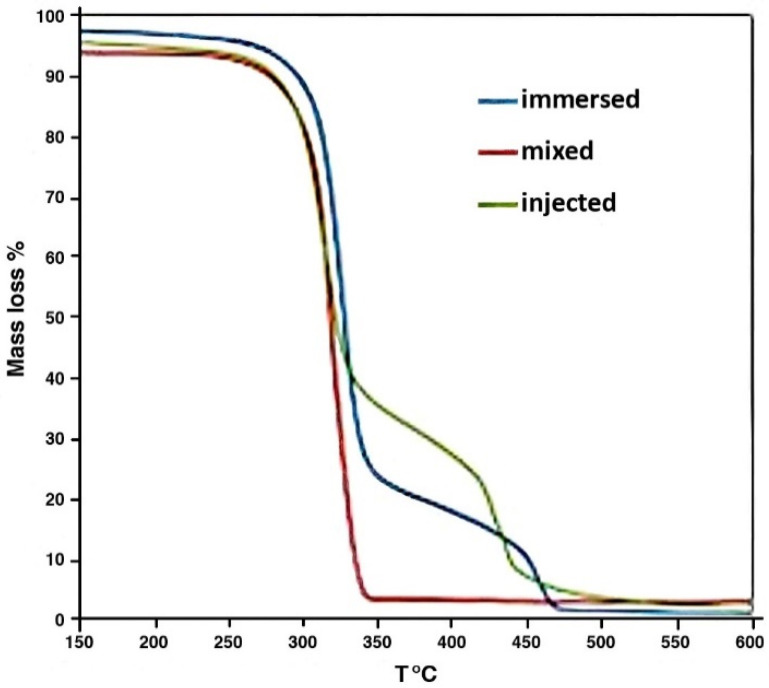
Mass loss (%) during heating of the foams in air.

In Figure 12, thermal decomposition of the foams is shown as the observed mass loss. Mass loss occurs when cellulose fibers start to oxidize; the kinetics of the oxidation depend on many parameters, including temperature, pressure, air flow, and the chemical properties of the cellulose material. In this particular case, where a foam composite is observed, it is more important to compare different materials.

All the foams were rather stable up to 250–270 °C and exhibited rapid mass loss in the range of 300–350 °C. However, it looks like the mass loss of the ANF-laden foams significantly slowed after 60–70%, and full decomposition was only achieved after 470–500 °C, which indicates the better temperature resistance of these ANF foams. The addition of ANFs to the foams has the effect of a slight increase in the foam decomposition onset, resulting in lower kinetics (mass loss rate). This indicates that the presence of ANFs does not deteriorate cellulose foam’s thermal stability—on the contrary, it may decrease the rate of thermal decomposition. It is necessary to examine this phenomenon in more details because of the possible differences in density and reactivity of the foams after ANF addition.

**Figure 13:**
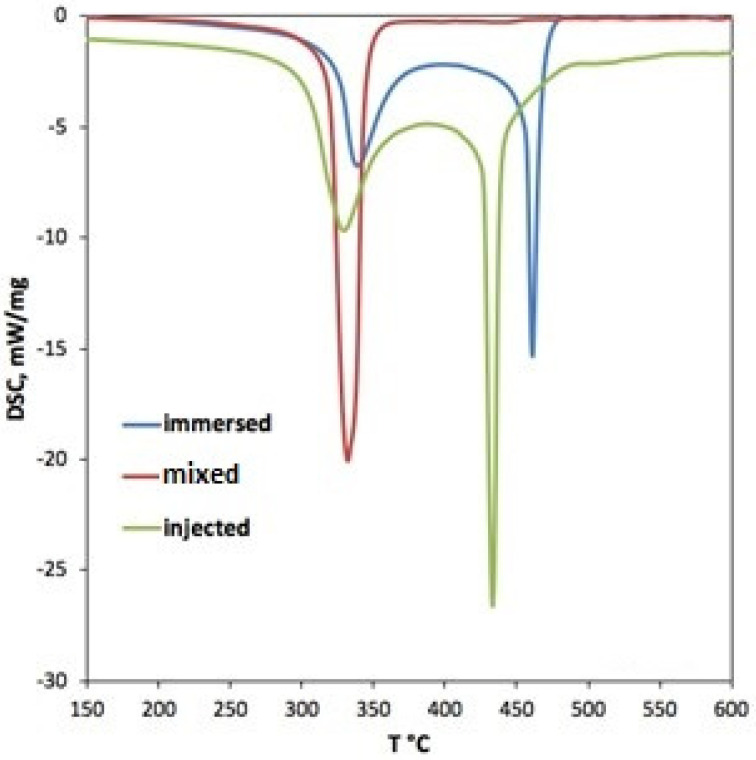
DSC traces for heat flux vs. temperature (exotherm is directed down).

DSC data obtained simultaneously with the TGA also clearly indicated distinct exothermic peaks related to the decomposition of the foams, as presented in Figure 13. Whereas the pure form eventually burns completely around 330 °C (onset of reaction at 320 °C, heat release of ~2120 J/g), the immersed ANF foam has two stages, at 325–360 °C and 450–500 °C, with heat release at about 1850 J/g. For the injected ANF foams, these stages are located between 330 and 350 °C and 425 and 440 °C. Notably, the mass loss rate and heat release are significantly lower than expected based on the mass fraction of ANFs (1 and 5%), confirming a positive impact of ANFs on the foam’s thermal resistance.

**Figure 14:**
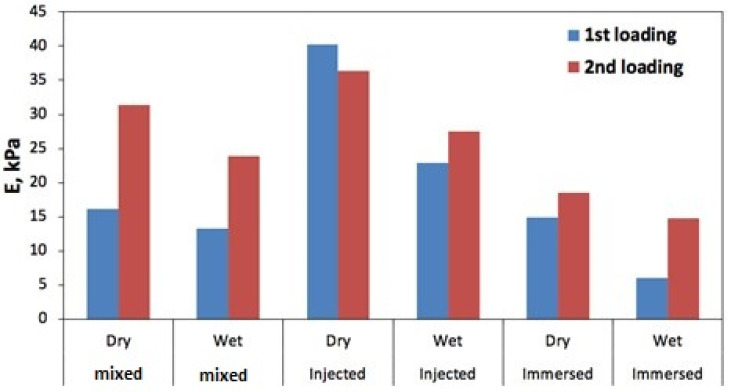
Estimated elastic modulus of the samples at 1st and 2nd loadings.

**Figure 15:**
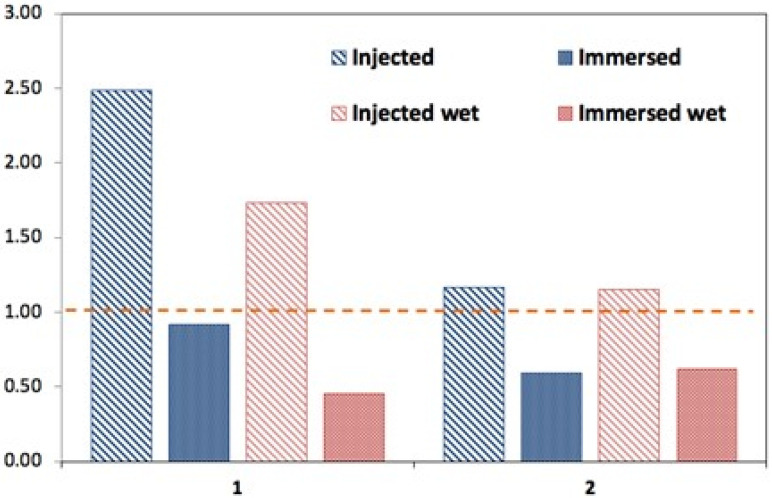
Relative changes in elastic moduli for the first- and second-loading foams. The horizontal line (equal unity) represents the ANF-free foam control sample.

For mechanical analysis in the creep mode, the estimated modulus was obtained from a non-linear fit of the stress/strain relation for every 30 s step, where the true logarithmic strain was used with the offset at every loading cycle. Figure 14 shows these values for the wet and dry samples. As the specimens might have internal structural deviations, these moduli were normalized to the original foam (without ANF) to capture the behavior of the trend (Figure 15). It is notable that the injected ANF foam maintains a higher elastic modulus (E) in both the dry and wet states, and also after the second (higher) loading, whereas the ANF immersed foam is inferior in this respect.

It might be recommended that the injection method is the best for introducing ANFs into cellulose foams, especially when high mechanical properties are desirable, as there is a more uniform distribution of nanofibers through the foam matrix. It might have slightly lower thermal stability vs. the immersed foam, but these differences are not highly significant, as presented in Figure 15.

## 5. Conclusions

This study presents a novel strategy for reinforcing cellulose fiber (CF) foam suspensions by incorporating alumina nanofibers (ANFs) with superior thermal properties. Given the crucial importance of good mechanical properties and thermal stability for the successful integration of CF foam composites into the packaging industry, understanding the effects of ANFs on the rheology and drainage and the dry foam’s mechanical and thermal properties is essential for achieving uniform properties of composite bio-based cellulose foam.

Rheological and mechanical analyses of CF foam composites revealed that the addition of lightweight and rod-shaped ANFs improves the rheological behavior of liquid foams and slows down drainage, which is critical for a good dry foam formation process and uniform porosity of the final foam product. Notably, higher ANF content in liquid foam delivers stability and uniform viscoelastic behavior during dry foam formation.

Dry cellulose foams reinforced with ANFs exhibited enhanced mechanical strength and thermal stability at high temperatures, confirming that the addition of lightweight metal oxide reinforcement increases temperature resistance. Various ANF incorporation techniques—co-mixing, immersion, and injection—were evaluated, with injection-based distribution proving to be the most effective in optimizing the foam properties.

These novel composite cellulose foams produced with the foam-forming method demonstrate strong potential for high-performance packaging and insulation applications across diverse temperature conditions, particularly relevant in climate change and extreme environmental stress conditions. In order to improve the processability of cellulose foam composites with ceramic nanofillers, it is necessary to investigate the resistance of cellulose–ANF foams to moisture, UV radiation, and mechanical stress, particularly in real-world conditions. Additional research should focus on optimizing ANF and other ceramic nanofiber incorporation methods to enhance performance while ensuring cost-effectiveness and environmental sustainability.

## Figures and Tables

**Table 1:** Samples that were prepared for the evaluation of liquid foams.

Liquid Foam	SDS/wt%	CF/wt%	ANF/wt%
Aqueous Foam	2	0	0
Fiber foam/Reference	2	20	0
Fiber ANF foam 1	2	20	2
Fiber ANF foam 2	2	20	5

**Table 2:** Labels and constituents of dry foams.

Dry Foam	SDS/wt%	Cellulose Fibers/wt%	ANF Fibers/wt%
Fiber ANF—foam mixed	2	20	5
Fiber ANF—foam immersed	2	20	5
Fiber ANF—foam injected	2	20	5

**Table 4:** Values of rheological parameters of foams with viscoelastic measurements.

Sample	Dynamic Yield Stress, τd^0^ (Pa)	Initial Shear Rate Viscosity, η_0_ (Pa·s)	k for *n* = 0.6 (Pa·s)	T for Static Yield Stress, τs^0^
Foam suspension	1.53	0.28	0.33 ± 0.03	60.2
Cellulose foam suspension	4.24	0.61	0.65 ± 0.03	64.8
Fiber foam with ANF1	6.72	0.65	0.71 ± 0.03	65.2
Fiber foam with ANF2	8.26	0.73	0.75 ± 0.03	66.5

## Data Availability

Data will be made available upon request.
